# Potential research ethics violations against an indigenous tribe in Ecuador: a mixed methods approach

**DOI:** 10.1186/s12910-020-00542-x

**Published:** 2020-10-17

**Authors:** Esteban Ortiz-Prado, Katherine Simbaña-Rivera, Lenin Gómez-Barreno, Leonardo Tamariz, Alex Lister, Juan Carlos Baca, Alegria Norris, Lila Adana-Diaz

**Affiliations:** 1 One Health Research Group, Faculty of Medicine, Universidad de Las Americas, Ecuador Calle de los Colimes y Avenida De los Granados, Quito, 170137 Ecuador; 2 Division of Population Health and Computational Medicine, University of Miami, Florida, USA; 3 Public Health Program, Faculty of Medicine, University of Southampton, Southampton, England; 4 Grassland Group, Technical University of Munich, Munich, Germany; 5 Ministerio de Biodiversidad, Quito, Ecuador; 6 Faculty of Psychology, Universidad de Las Americas, Quito, Ecuador

**Keywords:** Consent, Research ethics board, Indigenous populations, Waorani, Ecuador

## Abstract

**Background:**

Biomedical and ethnographic studies among indigenous people are common practice in health and geographical research. Prior health research misconduct has been documented, particularly when obtaining genetic material. The objective of this study was to crossmatch previously published data with the perceptions of the Waorani peoples about the trading of their genetic material and other biological samples.

**Methods:**

We conducted a mixed methods study design using a tailored 15-item questionnaire in 72 participants and in-depth interviews in 55 participants belonging to 20 Waorani communities about their experiences and perceptions of participating in biomedical research projects. Additionally, we conducted a systematic review of the literature in order to crossmatch the published results of studies stating the approval of an ethics committee and individual consent within their work.

**Results:**

A total of 40 men (60%) and 32 women (40%), with a mean age of 57 ± 15 years agreed to be interviewed for inclusion. Five main categories around the violation of good clinical practices were identified, concerning the obtention of blood samples from a recently contacted Waorani native community within the Amazonian region of Ecuador. These themes are related to the lack of adequate communication between community members and researchers as well as the voluntariness to participate in health research. Additionally, over 40 years, a total of 38 manuscripts related to the use of biological samples in Waorani indigenous people were published. The majority of the studies (68%) did not state within their article obtaining research ethics board approval, and 71% did not report obtaining the informed consent of the participants prior to the execution of the project.

**Conclusion:**

Clinical Research on the Waorani community in the Ecuadorian Amazon basin has been performed on several occasions. Unfortunately, the majority of these projects did not follow the appropriate ethical and professional standards in either reporting the results or fulfilling them. The results of our investigation suggest that biological material, including genetic material, has been used by researchers globally, with some omitting the minimum information required to guarantee transparency and good clinical practices. We highlight the importance of stating ethics within research to avoid breaches in research transparency.

## Introduction

The Amazon basin is home to many indigenous groups that live in a very traditional way, some of them in partial or complete isolation from the rest of the world [1–4]. Indigenous groups represent vulnerable populations from a research standpoint, and they often lack experience participating in research with communities being unaware of the research conducted in some cases.

Because of their lack of genetic admixture and lack of contact with the developed world, indigenous populations are unique for genetic and biomarker clinical research. Several studies have already documented research ethics violations among indigenous populations [5–13].

In 2012, the Waorani nationality, known as Nacionalidad Waorani del Ecuador (NAWE), brought to the attention the use of genetic samples to the government, to which the natives state that the samples were taken from the community without their consent [14]. The media attention and government support led to the creation of a commission to investigate these allegations. The objective of this study was to present available information on the case, which included a face-to-face field visit to the Waorani communities in the Ecuadorian Amazon region.

## Methods

### Study aim

The aim of this study was to conduct a systematic literature review on studies performed using biological material from Waorani natives and to analyse their perception about sample extraction and commercialization in the last 40 years.

### Study design

A mixed method analysis that used a quantitative approach through the use of a closed-questions 15-items tailored questionnaire and a systematic review of the literature concerning the use of biological samples among Waorani was employed for this study. The qualitative approach included in-depth interviews among the affected population.

### Setting

The study began on 2nd September 2012 with the in-situ visits to the Waorani territory. Interviews were completed on the 15th September 2012. The systematic review was finalised during early October 2019.

**Fig. 1:**
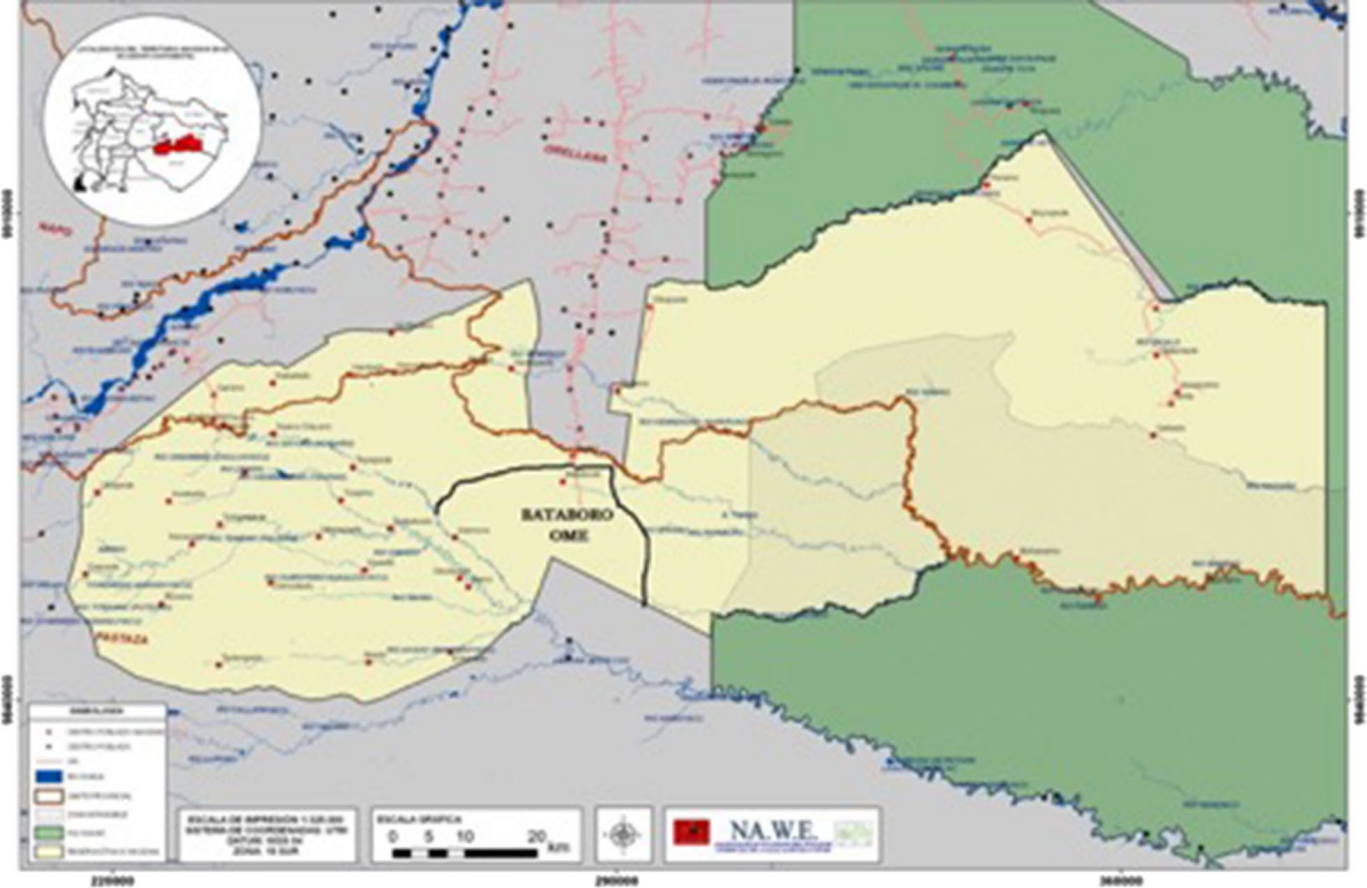
Waorani Communities located in the Ecuadorian Amazonian region. Dots represent the distribution of the indigenous families (this figure was created by Kawetipe Yeti president of NAWE for the purpose of this investigation and there is no copyright infringement or impediments for its use)

A total of 20 Waorani groups were visited in the three provinces that encompasses their territory in Ecuador, including the provinces of Pastaza, Sucumbios and Orellana (Fig. 1).

In order to access to the participants, the former president of the NAWE and two members of the community helped to locate the communities that were reported during the biomedical investigations.

### Study population

The Waorani are the most recently contacted and partially isolated indigenous group of the Ecuadorian Amazon rainforest. The name comes from the term "Wao", which means ‘people’ in the Wao-Terero language. Their territory currently covers more than 670,000 hectares, recognized by the Ecuadorian State. The Waorani are currently more than 3000 people grouped in 47 family groups distributed throughout the Waorani territory and the Yasuní National Park. Their territory runs between the Napo river to the north and the Curaray river to the south (Fig. 1).

### Subjects recruitment and sample size

Our sampling strategy consisted of a purposive sample strategy among the 47 Waorani groups located within their territory and included all the participants that fulfilled the inclusion criteria. 72 subjects completed the questionnaire and 55 of them agreed to be videotaped for the in-depth and personal interviews.

Twenty Waorani clans were visited per the published literature described below, obtained after reaching an agreement with the representatives of the NAWE. We focused on the older generations who were more likely to have repeated previous participation in clinical research helped by the former president of the NAWE and two members of the Waorani nationality.

### Ethical approval

The Ministry of Science and Technology (Senescyt) approved this study using an ad-hoc institutional review board including a board of Waorani tribesmen from NAWE to ensure all the ethical aspects are fulfilled. The informed consent was elaborated in Spanish and verbally translated to Wao-Terero through the Waorani members of the team. Since most of the participants did not know how to write or read, a Waorani translator was used to obtain consent from all the participants.

### Eligibility criteria

All subjects who were willing to contribute to the study and have completed at least 18 years of age at the time of the study were included. The subjects were relucted from the 20 communities described in the published scientific articles available after obtaining a written informed consent.

### Commission team

The *in-situ* commission team responsible for investigating the alleged bioethical violations of the Waorani rights was led by (EO) as Team Leader, Alegria Norris (AN) and Juan Carlos Baca (JCB) as Associate Investigators and Marta Carvajal (MC) as Team lawyer.

The commission was created under the direct request of the former President of Ecuador Rafael Correa Delgado and funded by the highest authority for science and technology of Ecuador (Senescyt). The in-situ commission included the president of the Waorani communities (NAWE) Cawetipe Yeti and two other members of the tribe.

### Qualitative methods

We used a qualitative approach to obtain in-depth information regarding previous experiences with research studies and biological samples collection. A videotaped interview was conducted by (EO) as the medical team leader, (JB) as the communication leader, (AN) as field expert and (MC) as the regulatory advisor. All the face-to-face interviews and responses were translated by one of the three Waorani guides in real-time while all the information was video recorded. The interview included open-ended questions regarding Waorani experiences when blood specimens were obtained in the past. Two authors (EO and LT) evaluated each comment, with disagreements resolved by consensus.

A content analysis was performed according to the information collected in the interview [15–17]. The purpose of the analysis was to identify Waorani experiences with research through the establishment of categories drawn from their discourse. Interview testimonies were coded by the team and compared for accuracy, with characteristic elements for each category grouped for analysis [18].

### Quantitative methods

We have developed a new survey (Additional file 1: Supplementary file 1) in coordination with a panel of experienced scientific researchers, NAWE representation and the staff of the commission (“Appendix”). The survey included 15 questions in the native Wao-Terero language. The domains of the survey included questions their experiences and attitudes about the use of research samples (“Appendix”). After we developed the survey, we pilot-tested it with the Waorani representatives (n = 6) for construct validity.

### Literature review

A systematic review of the literature of previously published studies who had collected data from one of the 20 identified Waorani communities.

**Fig. 2:**
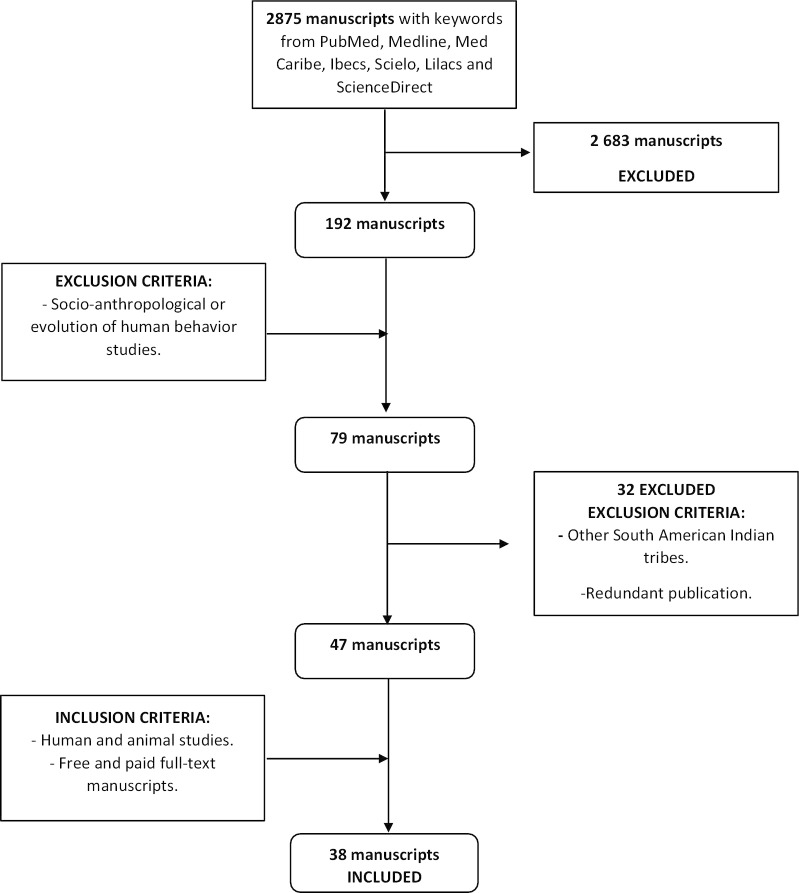
PRISMA chart of the searched manuscripts

The searching strategy encompassed the following databases: PubMed, Medline, Med Caribe, Ibecs, Scielo, Lilacs and ScienceDirect. The manuscripts were searched in either Spanish or English using the following terms: "Waorani", "Huaorani", "Guarani", "Auca", "Yasuní", "gen" and "genome". This review was designed to explore the groups which had conducted research on the native populations and through which scientific methodologies (Fig. 2).

### Statistical analysis

Pearson's chi-square statistic was used for categorical variables and a *t *test for continuous measures. Some continuous variables were not normally distributed, and therefore the Wilcoxon rank-sum test was used. To evaluate univariate differences, we reported the percentage of responses using the chi-square test. Analyses were performed using the statistical package SPSS (Chicago, IL), and all significance tests were two-tailed.

#### Results

### Baseline characteristics

The study consisted of 72 participants from 20 of the 47 communities, of whom 40 were men and 32 were women between 20 and 98 years, with a mean age of 57 ± 15 years consented for participation within this study. Of the participants, 97% had not received any formal education, with 1.4% having completed elementary school and 1.4% completing high school. In terms of language knowledge, 63.9% of participants understood no Spanish, with the remainder of participants having 12.5%, 5.5% and 18.1% of beginner, intermediate and fluent levels respectively. In the baseline characteristics of the included population by sex (Table 1), there were no significant differences on age, level of education and language by sex (p > 0.05).

**Table 1:** The relation between sex and age, level of education and Spanish level

Variables	Sex							
	Male n = 40			Female n = 32				
	M ± SD	n	%	M ± SD	n	%	X^2^	p
*Age*	58 ± 18			57 ± 18				
< 30 years (n = 5)		3	60.0		2	40.0		
31–50 (n = 31)		15	48.4		16	51.6	1.50	0.82
51–70 (n = 19)		11	57.9		8	42.1		
71–90 (n = 15)		10	66.7		5	33.3		
> 91 years (n = 2)		1	50.0		1	50.0		
Level of education								
Nonliterate (n = 70)		38	54.3		32	45.7	1.64	0.43
Elementary school (n = 2)		2	100		0	0		
Higher education (n = 1)		1	100		0	0		
*Spanish level*								
None (n = 46)		23	50.0		23	50.0	2.69	0.44
Beginners (n = 9)		6	66.6		3	33.3		
Intermediate (n = 4)		2	50.0		2	50.0		
Advanced (fluent) (n = 13)		9	69.2		4	30.8		

### Survey results

From the survey (Table 2), 65% of the participants state that they did not sign or agree with any informed consent when approached. Nearly all (96%) of the participants reported giving at least three blood samples for research, 29% of participants reported feeling pressured to provide samples and 75% of survey participants were given some level of explanation for blood donation. Nevertheless, participants state that 75% of these explanations were in a language other than their native tongue.

**Table 2:** Opened and closed questions

Questions		Sex			
		Male			Female
		n = 40			n = 32
		n	%	n	%
Q1. Have you had blood samples taken?	Yes	36	52.9	32	47.1
	No	4	100.0	0	0
Q2. How many times have you taken blood samples?	None	3	100.0	0	0
	1 to 2	29	52.7	26	47.3
	3 to 4	3	100.0	0	0
	> 5	1	33.3	2	66.7
	NK	4	50.0	4	50.0
Q3. Did you receive any kind of pressure or coercion for the extraction of blood samples?	Yes	11	52.4	10	47.6
	No	19	57.6	14	42.4
	NK	10	55.6	8	44.4
Q4. Did they explain the purpose of this in your language?	Yes	29	53.7	25	46.3
	No	9	56.3	7	43.8
	NK	2	100.0	0	0
What did you understand of the purpose?	Some diseases	26	59.1	18	40.9
	Medical checks	1	50.0	1	50.0
	Hepatitis	3	42.9	4	57.1
	Paludism	1	33.3	2	66.7
	NK	9	56.3	7	43.7
Q5. Did you sign any type of informed consent prior to the extraction?	Yes	8	66.7	4	33.3
	No	26	55.3	21	44.7
	NK	6	46.2	7	53.8
Q6. Was it national or foreign personnel that approached you for the study?	National	15	62.5	9	37.5
	Foreign	11	45.8	13	54.2
	Both	5	71.4	2	28.6
	NK	9	52.9	8	47.1
Q8. Do you know anyone who has had blood samples taken?	Yes	34	56.7	26	43.3
	No	0	0.0	0	0.0
	NK	6	50.0	6	50.0
Q10. Were there sick people in your community at the time of?	Yes	1	50.0	1	50.0
	No	18	56.3	14	43.8
	NK	21	55.3	17	44.7
Q11. Did the physicians return to perform some type of treatment related to the samples?	Yes	0	0.0	0	0.0
	No	38	55.9	30	44.1
	NK	2	50.0	2	50.0
Q12. Did they tell you that the samples were going to be moved out of Ecuador?	Yes	8	61.5	5	38.5
	No	28	51.9	26	48.1
	NK	4	80.0	1	20.0
Q13. Would you agree that your blood or a product of it is marketed by third parties?	Yes	0	0	0	0
	No	38	54.3	32	45.7
	NK	2	100.0	0	0
Q14. Do you think your rights were affected in any way?	Yes	31	54.4	26	45.6
	No	0	0.0	0	0.0
	NK	9	60.0	6	40.0

*NK* not know

From the open-ended questions, we found that some participants were willing to provide additional information. From the 72 subjects, 33% of recalled that the staff extracting the blood were foreigners, 56% reported that the medical personnel came from oil companies, and 17% reported being asked to provide samples by missionaries. Only 3% of the participants recalled someone being sick when the blood extraction was conducted. The vast majority (94%) recalled that the scientists or physicians never came back with the results of the samples or any treatments for ailments.

Most participants (75%) were unaware that their blood would be sent abroad for further analysis, and 79% believed their rights were infringed during the experiments. Ninety-seven percent of the subjects rejected the idea their blood could be shared or commercialized in any way, by any institution, in any country.

### Interview results

We interviewed 55 out of the 72 participants that were surveyed by our team (response rate 76.4%). The team grouped the coded segments in five categories as follow:

*Who was responsible for collecting the sample?*

This set of themes referred to the person who led the blood sample collection. The stories included both: national and foreigners medical professionals, missionaries, and colonist related to the oil company. During the initial contact during the late 50′s, the oil companies were the only non-indigenous people in the region. They provided some “support” to the indigenous communities in terms of medical attention and education.

In this category, 46 quotes were identified including some of the following examples, with the responses given in Wao-Terero translated to Spanish, then English:

*“The physicians that came to the community were from Ecuador and from other places, they used Dayuma to translate between us.”* Case ##03, Age group 71–90 yrs.

*“Someone translated, it was the Americans themselves who learned the language at the summer language institute (IVL), so they can study us."* Case ##02, Age group 51–70 yrs.

*How many times your blood was drawn?*

The domain in which the Waorani reported how many times they have given blood has shown that on average, the community members have experienced three blood extractions for research purposes in the last 40 years. This domain was found in 7 occasions and included testimonies like this:

*“Our blood was extracted at least two times. The first one around 1984 and a second time in 1991, with help of the missionaries.”* Case ##04, Age group 51–70 yrs.

*Voluntariness of the extraction*

This category referred to freedom in the decision-making process when natives gave their blood. The pressure or coercion arose from the entire community and from the research team. All cases reflect that they never signed any paper giving their consent either for the purpose mentioned or for other studies outside the country. The burden imposed on the tribe to offer the blood had nine quotes, including testimonies like the following:

*“We were concentrated in the Teweno Community [a community created by the missionaries and used as an indoctrination centre for the Waorani in the process of evangelization] with more than 100 other Waorani, more than 20 years ago, where we were allocated for the study” Case* ##50 Age group 51–70 yrs.

*“They told us that the doctors who were going to draw the blood came to our communities to see if we have Hepatitis A, B and C diseases. They took family by family. My daddy did not want to give blood, but the brother said yes, that he should go because it is a very serious illness and he could die”* Case ##19, Age group 51–70 yrs.

*Communication used during the studies:*

This category ranges from the language they used (Spanish or English instead of Wao-Terero) to the understanding of the commands that were given. The analysis of the interviews reflects that in some cases, the team leaders did translate part of the information to be given while in other cases, they did not.

*“We were moved in addition to other families towards the community of Teweno. The translator was not happy. We had some trouble with it."* Case ##02, Age group 71–90 yrs.

According to the Waorani, the understanding of the purpose for the visits was to see if their blood was good or bad, or to check for diseases in order to perform new treatments. No further information was given on the purpose of the studies.

*Returning with the results and final destination of the samples:*

This category reflects that researchers told the Waorani they will return to their communities with the results, nevertheless, in very few occasions they did but, in most cases, the Waorani reported they never came back.

*“When extracting the blood, they did not give us any information. We were transferred to Teweno, with the help of the missionaries” Case ##06, age unknown, estimated between 80–100 yrs.*

The Waorani state they did not know about the future of their blood, nor about the possibility of commercializing it for any economic value.

*“I knew they were going to investigate our diseases. Nevertheless, they never told me they were going to take the samples abroad. I did not agree with that, with selling the samples, we are human, I feel hurt, I didn't agree with that” Case ##57, age group 51–75 yrs.*

“No, they never came back, once they left, they never came back” Case ##57, age group 71–90 yrs.

### Use of samples for research

From our search, thirty-eight studies were identified to have carried out research on the Waorani population. A greater number of studies were carried out by international institutes, mostly by United States of America (USA) (n = 18; 47.4%), followed by European countries (n = 13; 34.2%), countries of Central and South America (n = 6; 15.8%) and Japan (n = 1; 2.6%) (Table 3).

**Table 3:** Chronological order of publications and details of Waorani’s research

No.	Institution's country	Author	Year	Number of population	Communities of origin	Study method	Material	Origin of samples	Ethics committee approval	Informed consent
1	USA	James W. Larrick et al. [28]	1978	600 Waorani	ND	Observational Epidemiological study	Surveys	–	ND	ND
2	USA	Jonathan E. Kaplan et al. [29]	1980	293 Waorani	Tiweno, Tzapino, Gabaro	Basic research, analytical procedures	Serologic test, skin test data and stool examination	Primary	ND	ND
3	USA	Jonathan E. Kaplan et al. [30]	1980	227 Waorani	Tiweno, Tzapino, Bai's, Gareno	Basic research, analytical procedures	Serologic test, skin test data and stool examination	Primary	ND	ND
4	UK	Theakston RD, et al. [31]	1981	223 Waorani	ND	Basic research, analytical procedures	Serologic test	Primary	ND	ND
5	USA	James W. Larrick et al. [32]	1983	227 Waorani	ND	Basic research, analytical procedures	Serologic test, skin test data and stool examination	Primary	ND	ND
6	USA	E.Wade Davis et al. [33]	1983	ND Waorani	ND	Observational Epidemiological study	Descriptive evaluation	–	ND	ND
7	UK	Theakston RD et al. [34]	1983	7 Waorani	ND	Applied Basic research—Animal study	Inoculation of human antibodies against the snake venoms	Primary	ND	ND
8	USA	James W. Larrick et al. [35]	1985	231 Waorani	Tzapino, Bai's, Tiweno, Gabaro	Basic research, analytical procedures	Serologic test	Primary	ND	ND
9	USA	C. Edward Buckley et al. [36]	1985	229 Waorani Other foreign groups	ND	Basic research, analytical procedures	Serologic test and skin test data	Primary	ND	ND
10	USA	James W. Larrick et al. [37]	1987	70 Waorani	ND	Basic research, analytical procedures	Serologic test	Primary	ND	ND
11	USA	David I. Watkins et al. [38]	1992	17 Waorani	ND	Basic research, analytical procedures	Serologic test	Primary	ND	ND
12	BRASIL	Fabrício R. Santos et al. [19]	1996	1 Waorani	ND	Basic research, analytical procedures	DNA sample	Secondary—Coriell Institute of Medical Research	ND	ND
13	USA	Evan E. Eichler et al. [20]	1996	1 Waorani	ND	Basic research, analytical procedures	Arrayed X-chromosome library	Secondary—Lawrence Livermore National Laboratory	ND	ND
14	GERMANY	Colm O’Huigin et al. [39]	1997	6 Waorani	ND	Basic research, analytical procedures	Serologic test	Primary	ND	ND
15	BRASIL	Fabrício R. Santos et al. [21]	1999	ND Waorani	ND	Basic research, analytical procedures	DNA sample	Secondary—National Institute of General Medical Science	ND	ND
16	USA	Michael A. Kron et al. [40]	2000	31 Dicaro Waorani 8 Non-Dicaro Waorani 16 Quichua	Dicaro	Basic research, analytical procedures	Serologic test	Primary	ND	Acquired
17	ECUADOR	Stephen R. Manock et al. [41]	2000	173 Waorani	15 Waorani Villages	Basic research, analytical procedures	Serologic test	Primary	Leadership of ONHAE	ND
18	BRASIL	Francisco M. Salzano [42]	2002	ND Waorani	ND	Systematic review	–	–	Local and National ethics committees	ND
19	USA	Lizhi Yu et al. [22]	2003	1 Waorani	ND	Basic research, analytical procedures	DNA sample	Secondary—Coriell Institute	ND	ND
20	JAPAN	Yasuhiro Go et al. [23]	2005	1 Waorani	ND	Basic research, analytical procedures	DNA sample	Secondary—Coriell Institute	ND	ND
21	MEXICO	Maria Mercedes Meza et al. [24]	2005	1 Waorani	ND	Basic research, analytical procedures	DNA sample	Secondary—Coriell Institute	ND	ND
22	USA	Eray Tuzun et al. [25]	2005	1 Waorani	ND	Basic research, analytical procedures	DNA sample	Secondary—Coriell Institute Cell Repository	ND	ND
23	ESPAÑA	Fabricio Gonzales [43]	2006	40 Waorani	ND	Basic research, analytical procedures	Serologic test	Primary	ND	Acquired
24	USA	Nicholas J. Marini et al. [26]	2008	ND Waorani	ND	Basic research, analytical procedures	DNA sample	Secondary—Coriell Institute Cell Repository	ND	ND
25	USA	Stephen Beckerman et al. [44]	2009	121 Waorani	ND	Observational Epidemiological study	Surveys	–	Institutional Review Boards of Pennsylvania State University and the University of Connecticut	Acquired
26	ESPAÑA	Fabricio Gonzales et al. [45]	2009	35 Waorani 102 Mestizos 102 Kichwa 102 Afro- Ecuadorian	ND	Basic research, analytical procedures	Serologic test	Primary	Metropolitan Hospital	Acquired
27	ESPAÑA	M. Baeta et al. [1]	2009	111 Waorani	Toñampare, Bameno	Basic research, analytical procedures	Saliva swab samples	Primary	ND	ND
28	USA	William Kuang-Yao Pan et al. [46]	2010	221 Quichua 99 Shuar 78 Huaorani 50 Cofan 31 Secoya	ND	Observational Epidemiological study	Surveys	–	University of North Carolina at Chapel Hill	ND
29	ESPAÑA	Luis Gomez—Perez et al. [47]	2011	36 Waorani	ND	Basic research, analytical procedures	Serologic test	Primary	University of the Basque Country	Acquired
30	GERMANY	Maria Geppert et al. [48]	2011	65 Waorani	ND	Basic research, analytical procedures	Buccal swab samples	Primary	Approved by an ethical commission not specified	Acquired
31	ESPAÑA	S Cardoso et al. [49]	2012	36 Waorani	ND	Basic research, analytical procedures	Serologic test	Primary	Institutional Review Board from UPV/EHU	Acquired
32	ESPAÑA	Luis Gomez—Perez et al. [50]	2013	36 Waorani Other foreign group	ND	Basic research, analytical procedures	Serologic test	Primary	Institutional Review Board from UPV/EHU	Acquired
33	GERMANY	Lutz Roewer et al. [51]	2013	40 Waorani Fifty different ethnic groups	ND	Basic research, analytical procedures	Buccal swabs, liquid saliva and capillary blood	Primary	Institutional review board of the Institute of Legal Medicine and Forensic Sciences, Berlin	Acquired
34	USA	Douglas S.London et al. [52]	2014	16 Waorani 63 Kiwchas	Kawymeno	Basic research, analytical procedures	Stool samples	Primary	ND	ND
35	USA	Douglas S.London et al. [53]	2015	121 Waorani 312 Kiwchas	Kawymeno	Observational Epidemiological study	Surveys	–	Institutional Review Board at Arizona State University	Acquired
36	UK	Massimo Mezzavilla et al. [54]	2015	22 Waorani 9 Kiwchas	ND	Basic research, analytical procedures	Serologic test	Primary	Institute of Legal Medicine and Forensic Sciences, Germany	Acquired
37	GERMANY	Maria Geppert et al. [27]	2015	20 Waorani 24 Kiwcha	ND	Basic research, analytical procedures	DNA sample	Secondary—obtained of other studies	ND	ND
38	ECUADOR	Edy Quizhpe et al. [55]	2016	199 Waorani	22 Waorani Villages	Observational Epidemiological study	Surveys	–	ND	ND

The table only described the indigenous communities belong to Ecuador. In the origin of the samples: primary refers to obtaining samples directly from the population and secondary refers to obtaining genetic samples from gene banks belonging to international institutions

*ND* not described

Eighty-nine percent of the published studies used Waorani blood samples 2,844 times between 1978–2016. Twenty-three percent of the studies reported obtaining genetic samples. The majority of the studies (84%) included authors from outside of Ecuador. Seventy-one percent of the studies used samples directly from the population (71%) for research after being collected, and 29% of the samples were obtained from biorepositories. The majority of the studies (68%) did not report obtaining research ethics board approval and 71% did not report obtaining the informed consent of the participants prior to the execution of the project.

Basic research studies aimed to analyze biological samples such as serologic test, skin test data and stool examination. The origin of the samples is classified into two types: primary, those that were obtained directly from the population (n = 22; 71%); and, secondary (n = 9; 29%) that were obtained from gene banks belonging to international institutions such as Coriell Institute, Lawrence Livermore National Laboratory, National Institute of General Medical Sciences or genetic samples obtained from other studies [19–27].

It is evident that the majority of the studies did not mention to get an informed consent of the participants before the commencement of the project (n = 27; 71.0%), neither ethics committee approval national nor international (n = 26; 68.4%).

## Discussion

This study reports the potential ethical violations from previous studies conducted on the Waorani natives in Ecuador. Based on the triangulation of the three sources of information collected in this study (literature review, survey and interviews) is clear that the Waorani tribe was not fully informed about the purpose of most of the studies, nor where the biological samples would end up.

Our study identified three major ethical violations, lack of informed consent, coercion and lack of return of results. The study documents among Waorani community members, with limited contact with what is known as “civilization”, who had blood samples taken without a fully informed consent process or documentation. It was reported that there was coercion by foreign staff from oil companies and missionaries for participation within studies and that there was a lack of return of results to the tribe, with samples reused for clinical research by foreign and national investigators. The strengths of this study include the mixed methods approach, which included structured face-to-face interviews with locals in their most comfortable language preference and the literature review of previous reports involving Waorani biological material.

The isolation of the Waorani tribes has made them highly coveted among scientists because of their unique genetic profiles and the lack of any human subject protection programs in the Ecuadorian state. Globally, indigenous people are underrepresented in genomic research studies for a variety of reasons including failure to engage indigenous communities in ethical and inclusive ways, lack of study transparency, and historical and recent research malpractice. These have sowed mistrust and justified peoples' unwillingness to share personal health information, including DNA, with the wider research community. Examples of research that involved large scale research misconduct and ethical violations include the Human Genome Diversity Project (HGDP) and the subsequent National Genographic Project. These projects began as endeavors to study worldwide human genetic diversity and global migration patterns, however, these projects failed to fully consider the damaging social and political implications to Indigenous communities. In the case of the HGDP, the project prompted strong resistance by the Indigenous Peoples' Council on Bio-colonialism and tarnished future trust in research and researchers [57]. The Havasupai Tribe filed a lawsuit against the Arizona Board of Regents over lack of informed consent and a violation of civil rights in addition to unapproved genetic research with DNA samples in prior research also. The case raised awareness of cultural and political sensitivities around what constitutes appropriate research [58]. These examples have raised concerns about the negative impacts that research harms (e.g., stigmatization, violation of individuals' rights, lack of benefit, and cultural incongruence) can have on Indigenous communities.

Potential explanations for our results include, firstly, within the timeframe when the initial publications were submitted, journals were not enforcing reporting research ethics board approval or informed consent use. The legal requirements required before obtaining a clinical research permit has changed in the last 50 years, however some basic precepts would seem that they have not been met. On the other hand, it is important to understand that at that time, Ecuador, lacked bioethical regulations and this could explain in part some of the scarcities in the revised investigations. At the same time, although it was evident that regarding human rights’ principles applied to medical investigations, some deficiencies were found, during the time of some of those investigations, The Belmont report, a US government commission document was not legally binding in the US, therefore not applicable to other parts of the world.

Second, poor communication abilities might cause that research brigades were offered as medical interventions, while in fact they were always intended to obtain biomedical data. Third, national and international scientists obtained blood samples from vulnerable populations and used it for research and commercial purposes.

The lack of evidence of proper ethical conduct in most of the published manuscripts and the lack of IRB permits including the informed consent process is worrisome. These flaws from the bioethical point of view are particularly important because indigenous populations are highly vulnerable due to several factors including poverty and high rates of illiteracy.

As local researchers, we found those practices are using a colonialist model, a visible practices found in developing and underrepresented populations through scientific malpractices [59]. According to the reflections of Restrepo & Rojas on the decolonial inflection, the coloniality of power can be said as the way to produce knowledge from a colonial model implies a place of power, exploitation and conflict [60]. From an expert position, foreign researchers and with the collaboration of Ecuadorians sold a health problem to the Waorani communities, used them to generate their scientific products without respecting the ethical parameters that they had in their societies [60].

There is a general ethical requirement that prospective participants in research give their voluntary consent to participate. Introducing coercion into this process breaks a critical step by affecting the voluntariness of consent and provides no reasonable alternative [61]. Coercion and undue influence has been a topic of recent attention when discussing payment to research participants since in most South American countries contrary to the United States, payment for research participation is not allowed [62]. However, in this case coercion was a threat rather than an inducement. During interviews, acts of coercion and the lack of informed consent were highlighted by some individuals in the Waorani communities. It was particularly troubling to hear that some community members felt healthcare personnel exerted pressure for everyone to provide blood samples and then never to return with the results of the exams or with treatments for ailments. This strengthens the theory that the reason for the blood draw was scientific, and not to treat any disease.

Another important result that deserves comment is the lack of return of results to research participants. With the recent changes to the common rule in the United States there has been a push to including some of this language in the informed consent in an effort to spark the conversation during the informed consent process. At the same time the majority of study participants will not only want to obtain results that are actionable but receiving all results. In this case, results were never returned.

One of the strengths of this study is the use of mixed methods, since it crosses the information of the scientific products with the testimonies, exploring the beliefs and perceptions of the same sample.

## Limitations

This study was not exempt from limitations. Many samples were taken several years ago and recall bias from the participants could have influenced our results. The lack of reporting of research ethics board approval in previous studies could be due to having no requirement to disclose this information at the time of publication. The translation of Wao-Terero to Spanish to English may also cause inaccuracies; however it was felt that this was a small limitation due to the review of the statements made by the participants by fluent Wao-Terero/Spanish speakers. These were subsequently translated into English, which was reviewed by fluent English speakers.

The content analysis was performed manually since no coding software was available at the time of the data collection. In this sense, we did not perform an inter-rater concordance test while reviewing the testimonies, instead we evaluate disagreement between items.

Recently, due to prior ethics violations on indigenous populations, a framework for research involving such communities has been proposed. The framework includes six principles when engaging in ethical research with indigenous people. The principles include understanding tribal sovereignty and research regulation, engaging and collaborating with tribal community, build cultural competency, improve the transparency of research practices, build tribal research capacity, disseminate findings in a community accessible format.

## Conclusions

We have documented for the first time the experiences and perceptions of indigenous vulnerable Waorani community members in the Amazonian region of Ecuador, about being approached by foreign and national scientists for the obtention of biological material. Upon interview and analysis of questionnaire results, several themes were highlighted, which included the negative experience the community members had with researchers, and the lack of a rigorous ethical research approach that the investigators presented. Clinical Research on the Waorani community in the Ecuadorian Amazon basin has been performed on several occasions. Unfortunately, the majority of these projects did not follow the appropriate ethical and professional standards in either reporting the results or fulfilling them. The results described that the lack of information concerning the approval of study, ensuring that participants understood the purpose of their samples being drawn was not correct. Although local regulations were lacking during the time of the study, international treaties, agreements and declarations were available worldwide, but not fulfilled by the majority of researchers involved in this study.

Although the government of Ecuador tried to further inspect some of these human rights violations, the investigations did not progress in courts. In this sense, this study aims to contribute to enhancing the awareness of unethical practices the occurred in Ecuador during the last 40 decades. Understanding the insights of this type of situations from a near-isolated communities in Ecuador, involving their experiences in previous medical research is a unique opportunity to raise conscience among the scientific community.

## Supplementary information

**Additional file 1**. 15-item questionnaire.

## Data Availability

The datasets used and analysed during the current study are available from the corresponding author on reasonable request.

## Abbreviations

HGDP: Human Genome Diversity Project
NAWE: Nacionalidad Waorani del Ecuador
ND: Not described
USA: United States of America
IVL: Summer Language Institute
