# Supplementary material for: Potential research ethics violations against an indigenous tribe in Ecuador: a mixed methods approach
Source: BMC Med Ethics. 2020 Oct 17;21:100. doi: 10.1186/s12910-020-00542-x (PMC7568418; doi:10.1186/s12910-020-00542-x)
Supplement: Supplementary file 1 — Additional file 1. 15-item questionnaire. [file 12910_2020_542_MOESM1_ESM.docx]

**Potential research ethics violations against an indigenous tribe in Ecuador: a mixed methods approach**

^1^Esteban Ortiz-Prado, ^1^Katherine Simbaña-Rivera, ^1^Lenin Gómez-Barreno, ^2^Leonardo Tamariz, ^3^Alex Lister, ^4^Juan Carlos Baca, ^5^Alegria Norris and Lila Adana-Diaz^6^

^1^One Health Research Group, Faculty of Medicine, Universidad de las Americas, Quito, Ecuador

^2^Division of Population Health and Computational Medicine at the University of Miami, Florida, USA

^3^Public Health Program, Faculty of Medicine, University of Southampton, Southampton, England

^4^Grassland Group, Technical University of Munich, Munich, Germany.

^5^Ministerio de Biodiversidad, Quito, Ecuador

^6^Faculty of Psychology, Universidad de las Americas, Quito, Ecuador

***Corresponding author:** Esteban Ortiz-Prado One Health Research Group, Universidad de las Americas, Quito, Ecuador Calle de los Colimes y Avenida De los Granados, Quito 170137, Ecuador. Email: e.ortizprado@gmail.comPhone: +593995760693

**15-item questionnaire for investigating bioethical sample collections among indigenous groups**

Q1. Have you had blood samples taken?

Q2. How many times have you taken blood samples?

Q3. Did you receive any kind of pressure or coercion for the extraction of blood samples?

Q4. Did they explain the purpose of this in your language?

Q4.1. What did you understand of the purpose?

Q5. Did you sign any type of informed consent prior to the extraction?

Q6. Was it national or foreign personnel that approached you for the study?

Q7. Do you know or recall some of the dates when the blood extractions were performed?

Q8. Do you know anyone who has had blood samples taken?

Q9. Was the location of the blood extraction unique? Or que community was moved somewhere else?

Q10. Were there sick people in your community at the time of?

Q11. Did the physicians return to perform some type of treatment related to the samples?

Q12. Did they tell you that the samples were going to be moved out of Ecuador?

Q13. Would you agree that your blood or a product of it is marketed by third parties?

Q14. Do you think your rights were affected in any way?

Q15. Would you agree if the government investigates these acts? and, if necessary, take any legal actions on your defense in national and international courts for not having obtained proper informed consent to yourself and the community?
